# An impact on medical student knowledge outcomes after replacing peer lectures with small group discussions

**DOI:** 10.15694/mep.2018.0000224.2

**Published:** 2019-05-21

**Authors:** Korakit Imwattana, Pattarachai Kiratisin, Patsharaporn Techasintana, Popchai Ngamskulrungroj

**Affiliations:** 1 Department of Microbiology; 2 Department of Microbiology; 3 Department of Parasitology; 4 Department of Parasitology

**Keywords:** Medical microbiology, Active learning, Small group discussion, Peer teaching

## Abstract

This article was migrated. The article was marked as recommended.

Active learning has been proven as an effective teaching method that increases students’ academic performance, satisfaction, and promotes life-long learning. A previous study showed that a peer lecture was time-consuming for both faculty members and students without any increase in knowledge outcome achievement of students when comparing to faculty lectures. Therefore, we replaced all peer teachings, taught in 2015, with faculty lectures and small group discussions in an organ-system part which focused mainly on applications of medical microbiology in 2016. The organ-system part was further divided into 3 sections according to type of organ systems. The knowledge outcome achievement was compared using MCQ scores. Peer lectures were mainly used as a teaching method only in 2015 (91.5%, 43 hours from a total of 47 hours) while none of them was used in 2016. On the other hand, SDG were mainly used as a teaching method only in 2016 (73.9%, 51 hours from a total of 69 hours). Students of 2016 had significantly higher average MCQ scores than those of 2015 only in the section 3 (80.8% as compared with 60.5%; p value < 0.001). There was no significant difference in section 1, section 2, and overall MCQ scores. Apart from routine teaching preparation, there was no out-of-class preparation time for faculty lectures and SGD while peer teaching required at least 0.5-2 hours of extra out-of-class preparation time from at least 1 faculty member and 10-12 students per 1 hour of teaching. In conclusion, SGD provided equal or more knowledge outcome achievement of the student with less time-consuming than peer lecture. Therefore, at least in our teaching environment, SGD was proved to be a better option than a peer lecture for teaching applications of medical microbiology.

## Introduction

Medical microbiology is a study of pathogenic microorganisms (bacteria, viruses, fungi, helminths and protozoa) and antimicrobial agents used against them ( Baig *et al.*, 2014). This subject poses great academic challenge as it contains a massive amount of basic knowledge as well as a wide range of clinical applications. This challenge makes conventional faculty teaching methods, which focus mainly on lower level of learning: memorize and understand, not adequate for effective medical microbiology teaching ( Southwick *et al.*, 2010).

In 2013, peer teaching method was implemented for medical microbiology teaching at the Faculty of Medicine Siriraj Hospital, Mahidol University, Thailand with a class of approximately 300 medical students. The teaching activity was conducted as a lecture competition performed by 12-14 student representatives (peer lecturers) which were trained for both microbiological knowledge and presentation skills prior to each lecture session. The best peer lecturer was decided by online votes from all students in the class. This teaching method improves students’ understanding of the subject and overall satisfaction without compromising the knowledge gain. ( Ngamskulrungroj *et al.*, 2017). However, it was still a one-way lecture that mainly focused on memorizing and understanding the subject and too time consuming for both students and faculty members ( Ngamskulrungroj *et al.*, 2017). In order to improve the higher level of learning, an additional teaching method was needed.

Active learning is a teaching method that involves students’ participation in class. Instead of sitting in a lecture hall receiving one-way information from lecturers, students contribute and gain new knowledge through various activities, e.g. group discussion, student debate, and project presentations ( Doody and Condon, 2012; King *et al.*, 2018; Mateo and Sevillano, 2018). Rather than giving lectures, teachers are required to facilitate class activities. This method of teaching is proven to not only improve students’ academic performances and satisfaction, but also to enable students to learn on their own and ultimately become effective life-long learners ( Azer *et al.*, 2013; Freeman *et al.*, 2014). However, as active learning focuses mainly on deep understanding, it typically requires more teaching time per learning outcomes than its counterpart; one-way spoon feeding large class lectures ( Phillips, 2005). Therefore, active learning is usually not an optimal method for teaching massive simple recall knowledge.

Small group learning is an active learning method which increases students’ participation by dividing students into smaller groups, which can take many forms. Among them, small group discussion (SGD) is a student-centered, problem-based learning method where students in the same group brainstorm and discuss the best answers to the given problems while their teachers act mainly as facilitators. SGD has been proven to improve students’ understanding on the subject as well as their satisfaction ( Annamalai, Manivel and Palanisamy, 2015; Hasamnis and Arya, 2017; King *et al.*, 2018).

During 2013 to 2015, teaching of applied medical microbiology were based solely on peer lectures. However, due to the disadvantages of the peer lectures as mentioned above ( Ngamskulrungroj *et al.*, 2017), we replaced them with the small group discussion in the class of 2016. Knowledge outcomes of students between the class of 2015 and 2016 were compared and reported.

## Methods

### A structure of medical curriculum

In Thailand, a medical student had to complete 6-year medical curriculum to get a degree of doctor of medicine. In our medical school, the 2 ^nd^ and 3 ^rd^ year was focused on basic medical sciences of normality and abnormality of human bodies, respectively. Therefore, a medical microbiology course, which was directly related to infectious diseases, was placed in the 3 ^rd^ year. The 3 ^rd^ year study was divided into two parts; ‘general concepts’ part and ‘organ systems’ part. The organ system part was also further divided into 3 sections: section 1 focusing on diseases of skin, musculoskeletal, and kidney-urinary-bladder system; section 2 focusing on cardiovascular, respiratory, gastrointestinal, and hepatobiliary system; section 3 focusing on nervous system, reproductive organs, organs of special sense, and systemic diseases. The separation and number of teaching hours of each sections were designated independently from this study by our medical school educational committee according to overall learning outcomes of Thai medical curriculum. For the medical microbiology course, the general concepts part focuses mainly on characteristics of each microbial pathogen, including growth, structures, morphology, virulence factors, pathogenesis, antimicrobial agents and principle of microbial investigations. The organ system part focuses on various applications of the basic knowledge from the concept part, including clinical, laboratory, and therapeutic approaches for infectious diseases. No patient was involved in the 3 ^rd^ year study.

### Implementation of active learning method in medical microbiology teaching

Small group learning has been proven to be an effective tool for teaching applications of knowledge. Therefore, small group discussion (SGD) was implemented in the organ system part, which focused on application of basic medical knowledge for approaches and managements of patient-based problems, of medical microbiology course in 2016. Moreover, we canceled all peer lectures, taught in 2015, as they were time-consuming for both faculty members and students without any increase in knowledge outcome achievement of students ( Ngamskulrungroj *et al.*, 2017). In 2015, each section began with peer/faculty lectures in a large classes followed by peer lectures in small classes. In 2016, each section began with faculty lectures followed by SGD. Table 1 compares teaching hours between the peer-lecture year (2015) and the SGD year (2016). Methodology of each teaching type was summarized in the table 1. Sample infectious cases for both lectures and SGD were designed and taught by collaboration of medical scientist and clinicians. The same example cases were used in both years.

In the year 2015, lectures in large classes were designed to introduce knowledge of general approach to infectious diseases and specific approach to patients with organ-specific infections for each organ systems. Approaches to common infectious diseases were given in more details during the lecture in small classes.

In the year 2016, faculty lectures included introduction to general approach to infectious diseases and specific approach to patients with organ-specific infections for each organ systems. SGD includes more detailed specific approaches to patients with organ-specific infections for each organ systems and approaches to common infectious diseases. As general approaches to infectious diseases were similar across all organ systems, only specific approaches to patients with organ-specific infections were required for the section 3 resulting in the minimal lecture hours of section 3. Therefore, the course was designed with a lower ratio of lecture/SGD in section 3 (2:21) comparing to those of the prior sections, section 1 (6:12) and section 2 (10:18),

In summary, apart from the canceling 43 hours of peer lectures, facultylectures were increased from 4 to 16 hours and 51 hours of SGD class were implemented in the class of 2016. This study was ethically approved by Siriraj Institutional Review Board under certificate number 589/2560 (Exempt).

### Assessment of knowledge outcome

Achievement of knowledge outcomes was evaluated by multiple-choice questions (MCQ) at the end of each section. MCQ of both years were optimized so that the difficulties and tested outcome were similar. A stem was typically an example of a patient problem. For example, “A 30-year-old man presented with watery diarrhea for 1 day. He had a history of traveling to India 5 days ago.” A question was one of the topic taught in each class ( table 1) including further Hx&PE, problem lists, laboratory tests and interpretations, definitive diagnosis, and treatment. For example, “What is the most appropriate laboratory investigation for this patient?” Five choices were given. Numbers of MCQ used in the year 2015/2016 were 30/35, 80/91, 50/26 questions for section 1, 2, and 3, respectively. Percentage of each MCQ answered correctly were collected as a MCQ score. Mean, standard deviation, and t-test statistic was done in Microsoft excel® 2010 licensed to Mahidol University. Chi square test was done by an online calculator ( https://www.socscistatistics.com/tests/chisquare/Default2.aspx) accessed in September 2018. Statistical significance was achieved at p-value < 0.05.

To minimize bias due to possible difference in the initial capability of students between each year, the MCQ score in each section in the year 2015 was normalized by multiplying with the ratio of “general concept” MCQ score of year 2016/2015.

**Table 1.** A comparison of teaching hours between peer-teaching and SGD year of teaching

Class type	Activities	Year 2015 by sections in hours (%)				Year 2016by sections in hours (%)			
		1	2	3	Total	1	2	3	Total
**Large class teaching (310-316 students)**									
Faculty lecture	A case-based lecture of approaches and managements of infectious diseases (10-15 minutes for each case) by faculty members. History and physical examination (Hx&PE) details were given followed by how to conduct further Hx&PE, to create problem lists, to choose laboratory tests, to interpret laboratory results, to make definite diagnosis, and to treat the disease. Each case were followed by a formative MCQ * answered by all students with electronic or colored-paper voter. Feedback was given immediately after each MCQ.	0(0)	2(9.5)	2(13.3)	**4** **(8.5)**	6(33.3)	10(35.71)	2(8.7)	**18** **(26.1)**
Peer lecture	Similar to the faculty lecture but taught by a group of 5-6 students (10-15 minutes for each case) followed by a short summary by faculty members (3-5 minutes for each case). For quality control, each student group had to practice with faculty members at least once (0.5-1 hour) prior to the lecture. One formative MCQ * and feedback were also done after each case.	6(54.5)	10(47.6)	5(33.3)	**21** **(44.7)**	0(0)	0(0)	0(0)	**0** **(0)**
**Small class teaching (25-28 students)**									
Peer lecture	Similar to the peer lecture in the large class.	5(45.5)	9(42.9)	8(53.3)	**22** **(46.8)**	0(0)	0(0)	0(0)	**0** **(0)**
Small group discussion	An example case was given to a group of 5 - 6 students to answered questions of further Hx&PE, problem lists, laboratory tests and interpretation, definitive diagnosis, and treatment (10 minute for each case), followed by a short lecture summary by faculty members (5-10 minutes for each questions). No formative MCQ * were used.	0(0)	0(0)	0(0)	**0** **(0)**	12(66.7)	18(64.3)	21(91.3)	**51** **(73.9)**
**Total**		11(100)	21(100)	15(100)	**47** **(100)**	18(100)	28(100)	23(100)	**67** **(100)**

* designed similarly to the other MCQ described in materials and methods

## Results/Analysis

### Sample size

Total of 615 medical students were included: 315 and 310 students studied medical microbiology in 2015 and 2016, respectively. No medical students were excluded from this study. According to Thai education regulation, student typically enters medical school right after finishing high-school. Therefore, student age of the 3 ^rd^ year medical student were largely 20-21 year old.

### MCQ scores

Students of 2016 had significantly higher average MCQ score than those of 2015 only in the section 3 (80.8% as compared with 60.5%; p value < 0.001). However, there was no significant difference in section 1, section 2, and overall scores ( table 2)

### Distribution of the teaching methods in each section

Peer lectures were mainly used as a teaching method in the year 2015 (91.5%, 43 hours from a total of 47 hours) and none of them was used in the year 2016. In fact, all classes in 2015 were taught by one-way lectures as the peer lectures was also considered one-way to the other students in the classes. On the other hand, SDG were mainly used as a teaching method in the year 2016 (73.9%, 51 hours from a total of 69 hours) and none of them was used in the year 2015. There were more SGD in the section 3 than those in the section 1 (66.7% VS 91.3%, p = 0.048, by Chi square test) and section 2 (64.2% VS 91.3%, p = 0.023, by Chi square test). Apart from a routine teaching preparation, there was no out-of-class preparation time for faculty lecture and SGD while peer teaching required at least 0.5-2 hours of extra out-of-class preparation time from at least 1 faculties and 10-12 students (1-2 groups) per 1 hour of teaching ( table 1). Details of the method distribution were showed in table 1.

**Table 2.** A comparison of mean MCQ scores (standard deviation) between the peer lecture year (2015) and the SGD year (2016)

	Section 1	Section 2	Section 3	Over all
2015	63.2 (23.6)	63.1 (21.1)	60.5 (19.5)	62.3 (21.0)
2016	54.8 (26.2)	68.2 (25.1)	80.8 (14.2)	67.3 (25.1)
P value	0.179	0.153	<0.001	0.058

Note: P value was calculated by unpaired 2-tailed T-test

## Discussion

Typically, active learning improves knowledge outcome ( Gilkar, Lone and Lone, 2016; King *et al.*, 2018) when comparing to one-way lecture, this is partially emphasized in our study that, at least, one section showed such improvement. However, it was surprising that, by measuring students’ knowledge outcome achievement, the other two sections in 2015, containing only one-way peer lectures, performed equally well to the other two in 2016, which mostly contained small group discussion. One possible explanation was that more SGD were used in the section 3. In addition, formative MCQ and feedback was completely omitted in the SGD. As feedback is a crucial part of active learning ( Phillips, 2005), such absence could somewhat reduce the performance of the SGD. However, as there was no formative MCQ and feedback in section 3 of the year 2016 either, such negative effect to the SGD could be minimal. Another more likely possibility was that each section contained different learning outcome depending on different system. Therefore, the positive effect of SGD might be system-specific and further study is needed.

Such increase in the knowledge outcome achievement did come with a cost, more teaching hours were required for the SGD year of 2016 when comparing to the peer lecture year of 2015 (67 VS 47 hours). However, as the extra out-of-class time was not needed for the SGD. In fact, approximately, twice the teaching time (94 hours) were actually required for the peer lecture to have at least equal knowledge outcome achievement. Therefore, this fact resulted in SGD became a less-time consuming method than the peer teaching.

Limitations of this study were as followed: firstly, as students also learned other subjects along with the microbiology course throughout the year, changes in other subject might influence microbiology scores. For example, a symptomatology subject, which taught general approach to patient, would directly influence how students approach patients with localizing infections; secondly, though normalized by the general concept scores, differences in non-microbiological knowledge and skills of the two group of students might also influence the results of this studies. For example, a recent study revealed that students with stronger critical thinking dispositions performed better in the problem-based learning process and achieved higher scores ( Pu *et al.*, 2019). Therefore, inclusion of measurements of these skills and knowledge are required for an unambiguous interpretation.

## Conclusion

In conclusion, SGD provided more or equal knowledge outcome achievement of the student with less time-consuming than peer lecture. Therefore, at least in our teaching environment, SGD is a better method than peer lecture for teaching an application of medical microbiology.

## Take Home Messages

• A small group discussion allowed students to achieve more or equal knowledge outcomes than or to a peer lecture for teaching applications of medical microbiology
• A small group discussion is less time consuming than a well-organized peer lecture

## Notes On Contributors

**Korakit Imwattana, M.D.,** An instructorDepartments of Microbiology, Faculty of Medicine Siriraj Hospital, Mahidol University. He was responsible for medical bacteriology teaching.

**Pattarachai Kiratisin, M.D. Ph.D.**, Professor and Director of the Institute for Technology and Innovation Management and Professor of Microbiology at Mahidol University. Pattarachai was a head of preclinical committee. He was responsible for medical bacteriology teaching.

**Patsharaporn Techasintana, M.D., Ph.D.**, Assistant professor at Departments of Parasitology, Faculty of Medicine Siriraj Hospital, Mahidol University. She is responsible for medical parasitology teaching.

**Popchai Ngamskulrungroj, M.D. Ph.D.**, Assistant professor and a quality manager of microbiology laboratory at Departments of Microbiology, Faculty of Medicine Siriraj Hospital, Mahidol University. Popchai was a committee member of 12 preclinical subjects. He was responsible for medical mycology teaching.
