# Supplementary material for: An impact on medical student knowledge outcomes after replacing peer lectures with small group discussions
Source: MedEdPublish (2016). 2019 May 21;7:224. Originally published 2018 Oct 2. [Version 2] doi: 10.15694/mep.2018.0000224.2 (PMC10704445; doi:10.15694/mep.2018.0000224.2)
Supplement: Peer review history

# Reviewer report: Reviewer response for version 2

**DOI:** 10.21956/mep.20235.r31357

**Published:** 2019-08-16

**Authors:** Richard Hays

**Affiliations:** 1 James Cook University

This review has been migrated. The reviewer awarded 3 stars out of 5 Thank you for the responses to my original concerns about version 1, and for the revision that addresses these. The result is an improved paper, although my concerns about peer lectures/peer teaching remain. If a student is provided with a presentation to deliver, then the outcome is likely to be that the student reads the slides, technically delivering the content but without the insights based on deeper knowledge and understanding. This may be no better (indeed possibly less useful) than allowing students to work through a presentation on their own. Good lecturing requires skills that a student is less likely to have. I also remain concerned about the evaluation by MCQ testing, as this may reflect only simple knowledge recall. I find it unsurprising that small group teaching produced better results, even by the methods used, but really do wonder why this model of peer teaching would even be considered. It is a long way from peer teaching methods used in most contemporary hybrid curriculum programs, where students leading discussions with access to other learning resources is more commonly seen.

Reviewer Expertise:

NA

No decision status is available

# Reviewer report: Reviewer response for version 2

**DOI:** 10.21956/mep.20235.r31358

**Published:** 2019-06-03

**Authors:** Simonetta Ausoni

**Affiliations:** 1 Department of Biomedical Sciences- University of Padova- School of Medicine

This review has been migrated. The reviewer awarded 4 stars out of 5 Thank you for working on a new version of paper, which, based on the suggestions of the reviewers, has actually improved the quality of the study

Reviewer Expertise:

NA

No decision status is available

# Reviewer report: Reviewer response for version 2

**DOI:** 10.21956/mep.20235.r31359

**Published:** 2019-05-21

**Authors:** Felix Silwimba

**Affiliations:** 1 University of Lusaka

This review has been migrated. The reviewer awarded 1 stars out of 5 this is a practical study in medical education. the findings are encouraging in support of student centered learning. I would have liked to learn more on qualitative issues of learners feelings about SGD.

Reviewer Expertise:

NA

No decision status is available

# Reviewer report: Reviewer response for version 2

**DOI:** 10.21956/mep.20235.r31360

**Published:** 2019-05-21

**Authors:** Muhammad Fazal Hussain Qureshi

**Affiliations:** 1 Ziauddin University

This review has been migrated. The reviewer awarded 4 stars out of 5 A very detailed and informative study. I would recommend people working in same discipline to read and conduct further research on this important aspect of learning medicine.

Reviewer Expertise:

NA

No decision status is available

# Reviewer report: Reviewer response for version 1

**DOI:** 10.21956/mep.18988.r27217

**Published:** 2018-10-29

**Authors:** Simonetta Ausoni

**Affiliations:** 1 Department of Biomedical Sciences- University of Padova- School of Medicine

This review has been migrated. The reviewer awarded 3 stars out of 5 I found that this is an interesting paper dealing with the intriguing issue of finding innovative strategies to teach basic sciences in the preclinical medical years. My general comment is that I am not surprised to see that the authors replaced peer lessons with small group discussions. In my view, peer lessons can be offered as pills, possibly provided by medical students of the 5th or 6th year or residents, but are not adequate to cover an entire course. In terms of examination scores, I think that comparing two cohorts belonging to different years has substantial limitation and is not very informative. My specific comment is that the paper would benefit from more detailed description of the methodological procedures used in the study. For example, the authors state that there was no out-of-class preparation time for faculty lectures and SDG. But SDGs are extremely complex to organize. How was the discussion planned? Did discussions involve clinicians and basic science teachers simultaneously? How did SDGs match with lessons? Why did the authors plan such a big discrepancy between section 3 (2 faculty lectures and 21 SGDs) and section 2 (10 lectures and 18 SGDs)? Was it done with any specific purpose? I think adding such information could provide an additional value to the manuscript.

Reviewer Expertise:

NA

No decision status is available

# Reviewer report: Reviewer response for version 1

**DOI:** 10.21956/mep.18988.r27215

**Published:** 2018-10-12

**Authors:** Richard Hays

**Affiliations:** 1 James Cook University

This review has been migrated. The reviewer awarded 2 stars out of 5 This is an interesting paper that may raise more questions than it attempts to answer. I work in a different context, where the idea of 'peer lectures', as I understand the term, would most likely not be acceptable. Could the authors please provide more information on what a peer lecture is? I am not a fan of lectures, unless they address topical, current or possibly contentious issues that can be clarified by someone who is relatively expert in the topic. Students are better at asking challenging and clarifying questions, and discussing issues to develop a shared understanding. While individual students may benefit from preparing a lecture, the recipients may be better off reading the literature themselves. Hence personally I wold always prefer to schedule small group discussions with students and, ideally, a faculty member. However, when it comes to the finding that SGD is better than peer lectures because of changes in examination scores from one year to the next, I am not sure that the authors have robust proof. Different cohorts, different learning experiences, different examinations etc mean that direct comparison is not really appropriate, particularly when the peer lectures were onlt 40-odd hours of the whole year. Care must be taken not to over-interpret data.

Reviewer Expertise:

NA

No decision status is available

# Reviewer report: Reviewer response for version 1

**DOI:** 10.21956/mep.18988.r27216

**Published:** 2018-10-05

**Authors:** Leila Niemi-Murola

**Affiliations:** 1 University of Helsinki

This review has been migrated. The reviewer awarded 4 stars out of 5 Thank you for the invitation to review this interesting article. It is sad to hear that the authors gave up the great idea of having peer lectures. That has been a great idea and, no doubt, preapring such leactures has been a great learning experience fot the students. However, as the authors noticed, the lecture is still rather passive learning for the other students. Thus, replacing the lectures with small group discussion is a pedagogically sound solution. Discussing clinically relevant problems will enhance the students problem solving skills, depending on the structure of the sessions. It will be interesting to know the next steps of this development.

Reviewer Expertise:

NA

No decision status is available
